# Fat deposition and growth performance in broiler chickens are diversely influenced by maize or wheat following dietary crude protein reductions plausibly involving insulin

**DOI:** 10.1186/s40104-025-01314-6

**Published:** 2025-12-24

**Authors:** Mengzhu Wang, Shemil P. Macelline, Sonia Yun Liu, Peter H. Selle

**Affiliations:** 1 Poultry Research Foundation Within The University of Sydney, Camden, NSW 2570 Australia; 2 School of Life and Environmental Sciences, Faculty of Science, The University of Sydney, Camden, NSW 2570 Australia; 3 Sydney School of Veterinary Science, The University of Sydney, Camden, NSW 2570 Australia

**Keywords:** Amino acids, Broiler chickens, Crude protein, Fat, Glucose, Insulin, Maize, Starch, Wheat

## Abstract

There is increasing interest in developing reduced-crude protein (CP) diets for broiler chickens because their commercial adoption would generate a diverse range of advantages that would enhance the sustainability of the chicken-meat industry. However, the development of reduced-CP broiler diets is proving to be not straightforward, particularly when dietary CP reductions exceed 30 g/kg. The capacity of broilers to accommodate dietary CP reductions when offered maize-based diets is superior to their counterparts offered wheat-based diets. Numerous factors could be contributing to this difference but have yet to be identified with certainty. Maize-based, reduced-CP diets characteristically support better weight gains and efficiencies of feed conversion than wheat-based diets, but this better growth performance is associated with increased fat deposition, monitored as heavier relative abdominal fat-pad weights. This is an intriguing dichotomy. Insulin is a powerful anabolic hormone in mammalian species capable of promoting fat deposition, protein accretion and growth, but the importance of insulin in avian species is usually dismissed. This is because broiler chickens are considered both hyperglycaemic and resistant to insulin. However, the likelihood is that young broiler chickens are more sensitive to insulin than is generally recognised and the anabolic properties of insulin may be contributing to the diverse responses observed between maize and wheat in the context of reduced-CP diets. Dietary CP reductions may trigger increased plasma ammonia concentrations and metabolic acidosis, but both factors can influence insulin secretion and insulin resistance. Maize has slower rates of starch digestion and glucose absorption than wheat and it has been suggested that this generates a more sustained insulin release resulting in increased weight gains and fat deposition. If so, this could be driving the differences generated by the feed grain selected as the basis of reduced-CP diets. The intention of this review is to explore this proposition because if the causal factors of the differences between maize and wheat can be identified the development and acceptance of reduced-CP broiler diets should be accelerated.

## Introduction

The successful development and adoption of reduced-crude protein (CP) diets for broiler chickens would generate a diverse range of advantages that would enhance the sustainability of the chicken-meat industry. Just one such advantage would be a reduced dependence on imported soybean meal as the principal protein source in broiler diets, which would apply to most countries around the globe, including Australia. However, the objective of achieving tangible dietary CP reductions without increasing fat deposition and compromising growth performance is not straightforward. Moreover, the identification of the critical impediments to the development of reduced-CP diets is proving to be a challenge.

The intensive genetic selection of broiler chickens over decades has led to outstanding advances in growth performance; however, this progress has led to some inadvertent and undesirable consequences including excessive fat deposition [1]. Fat deposition depresses feed efficiency, reduces meat yield and compromises meat quality in broiler chickens, none of which are conducive to sustainable chicken-meat production. It is becoming increasingly evident that reduced-CP diets amplify increases in fat deposition, which are readily monitored by determining relative abdominal fat-pad weights.

However, excessive fat deposition in broilers may has been recognised for decades. For example, Mabray and Waldroup [2] reduced amino acid levels from 120% to 70% of NRC requirements in 13.35 MJ/kg, maize-based diets offered to male birds to 57 d post-hatch. The amino acid reductions increased abdominal fat-pad weights by 48.2% (47.7 vs. 32.2 g) in absolute terms. Subsequently, Cabel and Waldroup [3] offered male broilers isocaloric, maize-based diets ranging in CP from 150 to 270 g/kg at 20 g/kg increments for 56 d. The decrease in dietary CP from 270 to 150 g/kg generated an increase of 94% (51.24 vs. 26.44 g) in absolute abdominal fat-pad weights and a two-fold increase in relative abdominal fat-pad weights from 11.8 to 22.8 g/kg.

The justification for the development of reduced-CP diets is compelling [4], but the objective is challenging, especially when dietary CP reductions exceed 30 g/kg. The outcomes of twelve published studies by the Poultry Research Foundation focusing on the development of reduced-CP broiler diets are tabulated in [5]. One remarkable, but consistent, outcome pursuant to dietary CP reductions is that broilers offered maize-based diets deposit more fat than their wheat-based counterparts, as declared by heavier relative abdominal fat-pad weights. Nevertheless, the growth performance of birds offered reduced-CP, maize-based diets is superior to their wheat-based counterparts. This is an intriguing paradox, which largely motivated this review, combined with the likelihood that lipids receive insufficient attention in broiler nutrition. This is emphasised in Liu et al. [6] in which responses to dietary protein concentrations in broiler chickens defined by growth performance, nutrient utilisation and carcass composition were modified by dietary lipid levels.

The Australian chicken-meat industry is heavily reliant on imported soybean meal from South America as the major source of protein and wheat is the dominant source of starch and energy in local broiler diets. Across seven Australian publications with wheat-based diets, a mean reduction in dietary CP of 32 g/kg (174 vs. 206 g/kg) allowed a formulated reduction in soybean meal inclusions of 57.4% (98 vs. 230 g/kg), but this compromised median weight gain responses by 9.17% and median FCR responses by 6.30% [7]. These data illustrate the potential of reduced-CP diets to restrict soybean meal importations, but clearly this would not be feasible if growth performance is compromised to these extents.

Broiler chickens are better able to accommodate dietary CP reductions in terms of growth performance when offered maize- than wheat-based diets. This was vividly illustrated in the direct comparison of Chrystal et al. [8] and supported by a second direct comparison [9] and the relevant outcomes are shown in Table 1. In the first comparison, male broiler chickens offered 165 g/kg CP diets, maize-based diets from 7 to 35 d post-hatch outperformed their wheat-based counterparts by 53.0% (2,370 vs. 1,549 g/bird) in weight gain and by 19.9% (1.473 vs. 1.840) in FCR. However, the maize-based diets supported heavier abdominal fat-pads by 70.7% (12.8 vs. 7.5 g/kg). In the second comparison the more modest dietary CP reduction from 220 to 180 g/kg compromised FCR by 3.51% (1.385 vs. 1.338) in birds offered maize-based diets, but this increased to 9.05% (1.433 vs. 1.314) with wheat-based diets. In terms of both fat deposition and growth performance, it is evident that there is a real dichotomy between maize and wheat in the context of reduced CP diets. Insulin is regarded as a powerful anabolic hormone in mammals, but the role of insulin in poultry is usually dismissed. However, as discussed later, young broiler chickens are probably more sensitive to insulin than is generally recognised. This review considers the possibility that the anabolic impacts of insulin are more evident in broilers offered maize-based than wheat-based diets, which fundamentally probably stems from the slower starch digestion rate of maize than wheat and the resultant impacts on glucose absorption along the small intestine and the pancreatic secretion of insulin in response.

**Table 1:** Effects of dietary crude protein concentrations on growth performance and relative abdominal fat-pad weights in broiler chickens offered either maize- or wheat-based diets from 7 to 35 d post hatch

Crude protein, g/kg	Growth performance, g/bird			Fat-pad weight, g/kg
	Weight gain	Feed intake	FCR, g/g	
Chrystal et al. [8]				
222 (maize)	2,214	3,208	1.453	6.4
193 (maize)	2,393	3,386	1.415	11.1
165 (maize)	2,370	3,481	1.473	12.8
222 (wheat)	2,403	3,487	1.453	6.4
193 (wheat)	2,386	3,507	1.471	8.5
165 (wheat)	1549	2,843	1.840	7.5
Greenhalgh et al. [9]				
220 (maize)	2,690	3,583	1.338	8.02
180 (maize)	2,598	3,586	1.385	10.16
220 (wheat)	2,720	3,574	1.314	6.19
180 (wheat)	2,510	3,595	1.433	7.78

Adapted from Chrystal et al. [8] and Greenhalgh et al. [9]

The genesis of this review stemmed from the consideration given to fat deposition in broiler chickens offered reduced-CP diets by Selle et al. [10] and the perception that the feed grain basis of reduced-CP diets modifies the anabolic impact of insulin put forward by Selle et al. [5]. In four studies then completed by Poultry Research Foundation involving maize-based diets a quadratic relationship (*r* = 0.763; *P* < 0.001) between dietary CP concentrations and relative fat-pad weights was detected. In four wheat-based studies there was a similar quadratic relationship (*r* = 0.682; *P* = 0.009). Instructively, the relevant regression equations predict that a dietary CP reduction from 220 to 170 g/kg with maize-based diets would generate an increase in fat-pad weights of 103.5% (12.33 vs. 6.06 g/kg). However, the same CP reduction with wheat-based diets would generate an increase in fat-pad weights of only 26.0% (8.25 vs. 6.55 g/kg). Thus, in broilers offered reduced-CP diets, maize-based diets supported 49.5% heavier-fat pads than wheat-based diets and this marked divergence requires an explanation.

If the causal factors of the differences between maize and wheat in the context of reduced-CP diets can be identified, then the development and acceptance of reduced CP, wheat-based diets should be expedited. This holds relevance as wheat is the dominant feed grain for chicken-meat production in Australia, but also in Canada and parts of Europe. The intention of this document is to review the impacts of starch and protein on lipid metabolism in broiler chickens with an emphasis on lipid deposition and relative abdominal fat-pad weights when broiler chickens are offered reduced-CP diets based on either wheat or maize.

That reductions in dietary CP concentrations generate increases in abdominal fat deposition in broiler chickens is evident in a meta-analysis [11]. In this meta-analysis there was a significant relationship (*P* < 0.001) between dietary CP and relative abdominal fat-pad weights such that a 10 g/kg CP reduction would cause an 8.8% increase in fat-pad weights. Unfortunately, the dietary compositions in this meta-analysis were not stated.

**Fig. 1:**
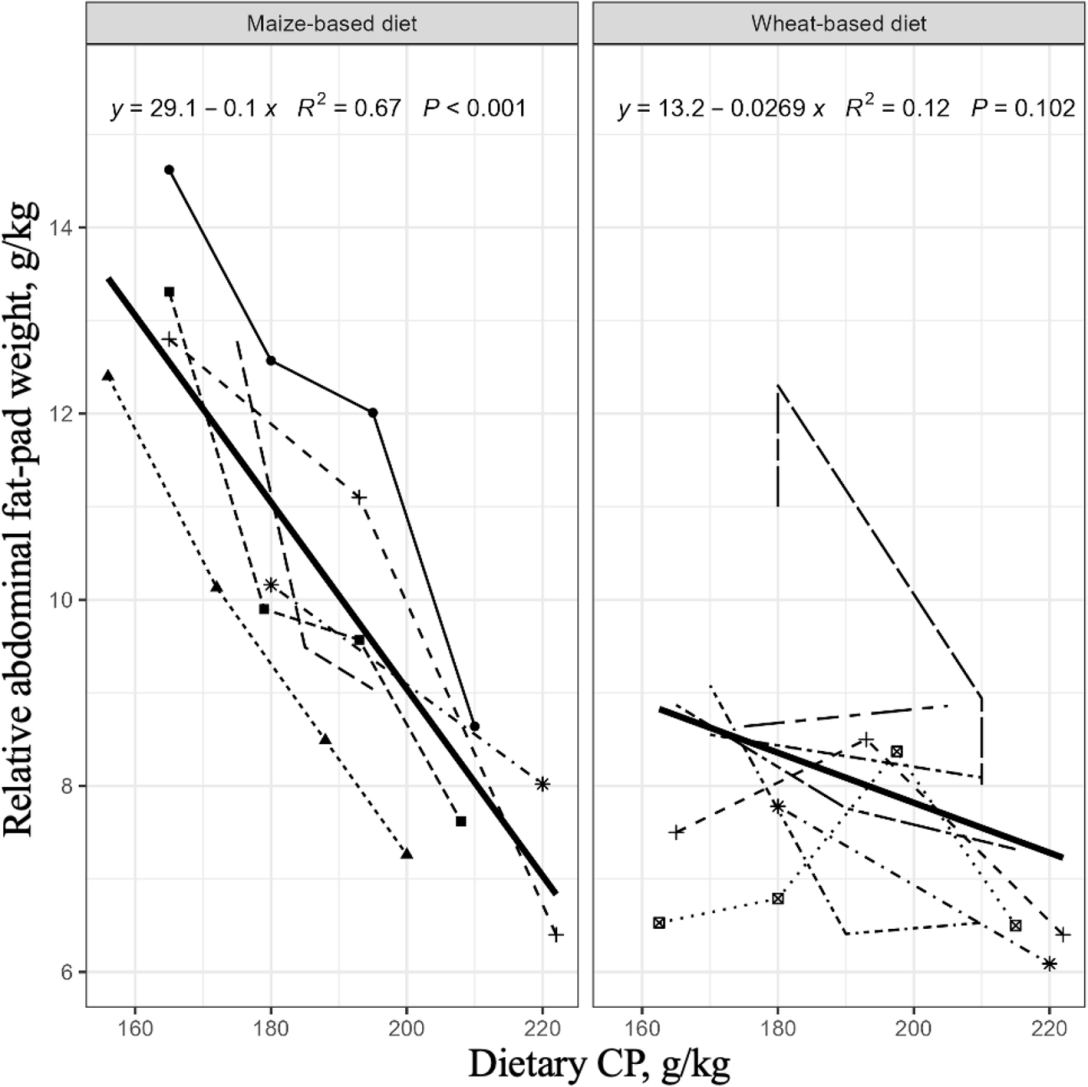
Correlations between dietary CP concentrations and relative abdominal fat pad weights in male broiler chickens offered maize- or wheat-based diets, where the relationship for maize (*P* < 0.001) is significant but wheat (*P* = 0.102) is not significant. Based on data in Tables 1 and 2 in Selle et al. [5]

The outcomes of six reduced-CP feeding studies (20 data points) with maize-based diets and eight studies (23 data points) completed with wheat-based diets are tabulated in Selle et al. [5]. It was deduced from the tabulated data that a 30 g/kg dietary CP reduction would be associated with a 15.6% (416 vs. 351 g/kg) increase in analysed dietary starch concentrations, a 2.6-fold (27.0 vs. 10.4 g/kg) increase in non-bound amino acid inclusions but a 40.4% (140 vs. 235 g/kg) decrease in soybean meal inclusions. Thus, even reasonably moderate reductions in dietary CP generate profound changes in the starch and protein constituency of broiler diets. However, as discussed, birds offered maize-based diets are better able to accommodate dietary CP reductions than birds offered wheat-based diets. It also became evident that birds offered maize-based diets have a greater propensity to accrue heavier abdominal fat-pads following dietary CP reductions than birds offered wheat-based diets. This is illustrated in Fig. 1, which is based on the tabulated data in [5]. There is a highly significant linear relationship (*r* = 0.671; *P* < 0.001) between decreasing dietary CP and increasing relative abdominal fat-pad weights in birds offered maize-based diets. In contrast, the same relationship does not approach significance (*r* = 0.346; *P* = 0.102) in birds offered wheat-based diets. In addition, it may be deduced from the same data that an average dietary CP reduction of 41.1 g/kg (213.0 to 171.9 g/kg) in wheat-based diets generated a 20.4% (8.92 vs. 7.41 g/kg) increase in relative abdominal fat-pad weights. In contrast, a similar dietary CP reduction of 41.5 g/kg (209.2 to 167.7 g/kg) in maize-based diets generated a three-fold larger increase of 61.9% (12.68 vs. 7.83 g/kg) in relative fat-pad weights.

The quantities of vegetable oil and/or animal fats that are included in broiler diets are modest in comparison to dietary concentrations of starch and protein. Thus, the increases in lipid deposition, as reflected in relative abdominal fat-pad weights, following CP reductions are indicative of de novo lipogenesis [12], which are more evident in maize-based diets.

## Macronutrients: Fats, protein, starch and their digestive dynamics

Instructively, Rérat [13] issued the caution that “the study of kinetics associated with differences in the relative absorption rate of the various nutrients is necessary for a fuller understanding of variations in the nutritive value of feeds”. Therefore, it is relevant that nearly three decades later Pedersen et al. [14] considered the impact of feed ingredients in complex diets on protein (N) and starch disappearance rates in broiler chickens. A combination of full-fat soy and soybean meal decreased the protein (N) digestion rate by more than 20% compared with diets containing either feedstuff individually; thus, different feed ingredients in complex diets influence nutrient digestive dynamics.

In reality, ‘digestive dynamics’ is a truncation as it extends to embrace the digestion of nutrients, intestinal uptakes of the end-products of digestion, their transition across small intestinal enterocytes, entry into the systemic circulation and, ultimately, their incorporation into skeletal muscle to drive growth which requires an energy input of 5.35 kJ for the accretion of 1 g of protein in broiler chickens [15]. Therefore, appropriate provisions of non-protein energy and amino acids at sites of protein synthesis are crucial for efficient muscle accretion and rapid growth [16]. The nutritional dynamics of protein and amino acids in relation to performance, health, welfare, and cost of production were recently reviewed by Adhikari et al. [17]. However, our contention is that if reduced CP diets are to be developed successfully, in addition to individual considerations, the interactive nutritional roles of fats, proteins and starch will need to be considered collectively. The next three sections of this review will address some relevant aspects applicable to fats, protein and starch on an individual basis.

That the nutritional roles of fats, proteins and starch should be considered collectively gains support in two papers by Liu et al. [6, 18]. The Liu et al. [18] experiment used a right-angled triangle mixture design, with 14 diets with energy densities of 13.60 MJ/kg, but the energy was derived from different dietary concentrations of starch, protein and lipids. The macronutrients were expressed as energy derived from starch, protein and fat and the dietary treatments were offered to male Ross 308 birds from 10 to 23 d post-hatch. The outcomes indicated that energy derived from lipid had the greatest impact on feed intake and increasing protein-derived energy increased weight gain, but energy derived from starch and lipid had little impact on weight gain. Efficiency of feed conversion may be improved by either increasing protein energy intake or decreasing starch energy intake and a balance between protein and energy intakes is required for efficient muscle protein deposition.

**Fig. 2:**
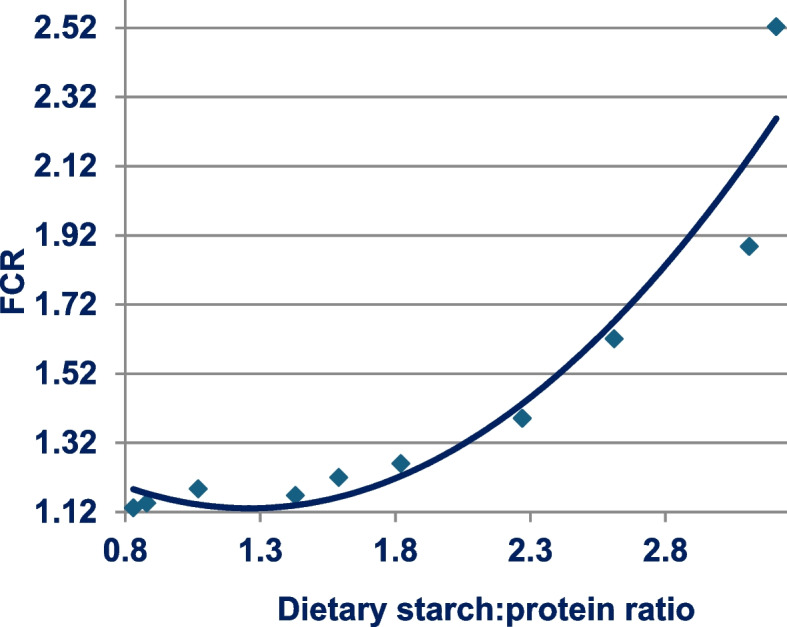
Quadratic relationship (*r* = 0.957; *P* < 0.0001) between analysed dietary starch:protein ratios and FCR where *y*_(FCR)_ = 1.599 − 0.744 × ratio + 0.296 × ratio^2^. Adapted from Liu et al. [6]

In the Liu et al. [6] study, 10 dietary treatments with identical energy densities and protein concentrations ranging from 154 to 400 g/kg and two lipid levels of 46 and 85 g/kg were offered to male Ross 308 broilers from 7 to 28 d post-hatch. Predictably, the effects of dietary protein concentrations on broiler performance were profound; however, the impact of dietary protein on performance in broiler chickens was modified by dietary lipid concentrations. In this study, ‘diet 9’ was atypical in that it contained 168 g/kg soy protein isolate, 139 g/kg soybean meal and 555 g/kg maize. Analysed concentrations of protein (N), starch and fat in this diet were 322, 344 and 94.6 g/kg, respectively. However, this diet bettered 2014 Aviagen performance objectives by 16.0% (1,609 vs. 1,387 g/bird) in weight gain and by 19.7% in FCR (1.187 vs. 1.479) post-hatch. An FCR of 1.187 from 7 to 28 d post-hatch is remarkable and emphasises the potential of modern genotype broiler chickens that is not being realised by more conventional, cost-effective diets. Instructively, there was a significant quadratic relationship (*r* = 0.957; *P* = 0.0001) between analysed dietary starch:protein ratios and FCR in this study as illustrated in Fig. 2. The quadratic regression equation predicts that a dietary starch:protein ratio of 1.26 would support an FCR of 1.131, which represents an improvement of 23.5% relative to Aviagen performance objectives.

Finally, feedstuffs with predetermined starch and protein digestion rate constants were formulated into six diets with similar amino acid and energy densities, that were offered to broilers from 7 to 35 d post-hatch in Liu et al. [19]. Starch:protein digestion rate ratios ranged from 1.567 to 2.453 and it was found that a ratio of 1.663 supported the optimal FCR of 1.450. Retarding starch digestion rates, accelerating protein digestion rates or condensing starch:protein digestion rate ratios improved efficiency of feed conversion across all dietary treatments. In short, this experiment demonstrated the potential of incorporating starch:protein digestive dynamics into the least-cost formulation of broiler diets. The two optimal dietary starch:protein ratios mentioned above are quite different; however, the optimal ratio is not a constant and will inevitably vary with different dietary treatments. More aspects pertaining to digestive dynamics in poultry may be gleaned from the reviews [20–23]. In the interests of brevity, these reviews are recommended for those interested in digestive dynamics.

## Fats

Fats (and/or lipids) have not received the same level of attention as proteins and carbohydrates in poultry nutrition and yet the relevant pathways involved in their digestion, absorption and metabolism are more complex. This is illustrated in the following sentence from Krogdahl's review [24] of the digestion and absorption of lipids in poultry: “Ingested lipids undergo intestinal emulsification, digestion, micellar solubilization, cell membrane permeation, intracellular esterification and incorporation into lipoproteins before release to the interstitial fluid”. Two thorough reviews have been subsequently published. Ravindran et al. [25] considered the digestive physiology of fats and factors influencing their utilisation in poultry nutrition and Wang et al. [26] discussed the factors affecting adipose tissue development in broiler chickens. Additional reviews with relevance to various aspects of fats in poultry nutrition include those by [27–32].

The caveat was issued by Kim and Voy [33] that broiler chickens accumulate more adipose tissue than it is physiologically necessary due to inadvertent consequences of selection for rapid growth. Importantly, Choct et al. [34] noted that excess fat deposition depresses efficiency of feed conversion. Thus, the problem of excess fat deposition is only exacerbated in birds offered reduced-CP, maize-based diets.

Fats possess the highest energy density across the three macronutrients in poultry diets as the energy densities of fats, starch and protein are approximately 34.31, 16.69 and 15.51 MJ/kg, respectively [35]. Dietary fat concentrations are derived from their intrinsic presence in feedstuffs coupled with dietary additions of ‘fats’ per se. As an example, Chrystal et al. [8] offered broilers diets based on either maize or wheat with an energy density of 12.85 MJ/kg and a CP content of 222 g/kg from 7 to 35 d post-hatch. The maize-based diet contained 35 g/kg soy oil and a calculated crude fat content of 85 g/kg and the wheat-based diet contained 52 g/kg soy oil with a crude fat content of 93 g/kg. Thus, the added vegetable oil constituted approximately 9.3% and 13.9% of the energy density in the maize and wheat-based diets, respectively. Therefore, additional fats are required to meet the targeted energy density of poultry diets, which may exceed 13.0 MJ/kg for older broiler chickens.

The fatty acid compositions of commonly used animal fats and vegetable oils are tabulated in Ravindran et al. [25]. Examples include beef tallow, which contains saturated long-chain acids, coconut oil, which is rich in saturated medium-chain acids, and safflower oil in which unsaturated long-chain acids are dominant. The energy generated by these three fat sources in poultry was compared [36]. The average total metabolisable energy (TME) at −1-, 3- and 10-d post hatch was 32.79 MJ/kg for coconut oil, 33.21 MJ/kg for beef tallow, and 34.86 for safflower oil. The mean metabolisability (TME/gross energy) coefficients were 0.885, 0.891 and 0.894 for coconut oil, beef tallow and safflower oil, respectively.

### Fat digestion

Fat digestion takes place mainly in the jejunum (70% to 87%) and, to a lesser extent, the ileum (6% to 26%) in broiler chickens [31]. The digestion of fats in broiler chickens is mainly achieved by pancreatic lipase (triacylglycerol lipase) and lipase activity in pancreatic secretions in broiler chickens increases sharply, by 52.8%, immediately after feeding [37]. Fats and lipids are made up of triacylglycerol, which are hydrolysed by pancreatic lipase to generate one monoglyceride and two fatty acid molecules, which can be absorbed along the small intestine. The presence of the protein coenzyme, co-lipase, is essential for optimal lipase activity. The digestion of fats is facilitated by the grinding action of the gizzard coupled with episodes of reverse peristalsis and by the hepatic secretion of bile, which include bile salts and phospholipids and results in fat emulsification. Bile salts emulsify large fat globules into tiny droplets, thereby increasing the surface area to facilitate the access of lipase to its substrate. The end-products of lipolysis are incorporated into mixed micelles with bile acids and these micelles significantly enhance solubility of lipids in the intestinal aqueous environment. The hepatic secretion of bile must be adequate as bile is essential to both emulsification and micelle formation in the intestine. The importance of bile salts in fat digestion was demonstrated by Krogdahl [24]. Across six studies, broiler chickens were offered diets containing an average of 106 g/kg fat (tallow or an animal blend). At an average inclusion of 3.60 g/kg, the addition of bile salts to these diets improved fat digestibility by 7.42% (0.796 vs. 0.741).

Exogenous phytase is now a ubiquitous inclusion in broiler diets globally. Therefore, it is relevant that 500 FTU/kg phytase increased apparent ileal fat digestibility by 4.16% (0.827 vs. 0.794; *P* = 0.001) in broilers offered both maize- or wheat-based diets in [38]. Phytase also significantly increased average digestibilities of selected, saturated (4.59%) and unsaturated (4.18%) fatty acids to similar extents. One possible explanation for these responses was initially advanced by Ravindran et al. [39]. The prior hydrolysis of dietary phytate by exogenous phytase in the gizzard may partially prevent the formation of metallic soaps in the small intestinal lumen. Metallic soaps can arise from interactions between fatty acids and metal cations, including calcium, and are essentially insoluble and non-digestible and interfere with fat digestion.

As reported by Tancharoenrat et al. [40], the apparent fat digestibility coefficient of soy oil was negative in the duodenum and ranged from 0.29 in the proximal jejunum to 0.82 in the distal ileum. The digestibility of tallow fat was again negative in the duodenum and ranged from 0.32 to 0.74 in the jejunum to the ileum. Negative apparent fat digestibility coefficients in the duodenum are indicative of endogenous fat secretions into the gut lumen. The main sources of endogenous fats are bile and desquamated intestinal epithelial cells [25]. For individual fatty acids, distal ileal apparent digestibility coefficients were 0.77 for stearic acid (C18:0), 0.85 for palmitic acid (C16:0), 0.90 for oleic acid (C18:1) and 0.94 linoleic acid (C18:2) in soy oil. The corresponding outcomes for fatty acids in tallow were 0.58 for stearic acid, 0.68 for palmitic acid and 0.90 for both oleic and linoleic acids in the same study. The conclusion of Tancharoenrat et al. [40] was that both degree of saturation and chain length influenced the digestibility of fatty acids with better digestion of unsaturated fatty acids and short-chain fatty acids than their saturated and long-chain counterparts.

### Fat absorption

The absorption process constitutes the most limiting step in fat utilisation in poultry, regardless of the dietary fat source, was the view expressed by Rodriguez-Sanchez et al. [31]. These researchers also found that the absorption of unsaturated fat was more efficient and faster than saturated fat and the capacity of chickens to absorb fat increased with age. The duodenum is the main site of fat digestion and the jejunum the primary site of fat absorption. The formation of mixed lipid-bile salt micelles is critical to the intestinal uptake of monoacylglycerol and fatty acids and unsaturated fatty acids. The micelles are water soluble aggregates of lipid molecules and can be passively absorbed by the gut mucosa. Within enterocytes, glycerol and fatty acids are re-esterified, incorporated into chylomicrons and enter the lymphatic system [25].

### Fat transition across the gut mucosa

The absorption of dietary fat and its metabolism in enterocytes was reviewed from the human perspective by Carreiro and Buhman [41]. The digestive products of dietary fat are reconstituted into triacylglycerol within the enterocytes of the small intestine. Then the triacylglycerol is incorporated into chylomicrons and secreted into the lymph circulation or incorporated into cytoplasmic lipid droplets. Additionally, fat digestive products may serve as an energy source, signaling molecules or be incorporated into other complex lipids. The oxidation of amino acids, glucose, and fatty acids as energy substrates within avian enterocytes was investigated [42]. Propionate (2.74) and butyric (2.20) acids were oxidised to limited extents, but easily exceeded by glutamate (93.9) and glucose (22.7), while intermediate substrates included aspartate (17.6), glutamine (15.3) and alanine (9.41). The numbers in parentheses represent the average amount of energy substrate oxidised to CO_2_ by broilers at 7, 21 and 42-d post-hatch expressed as nmol CO_2_/10^6^ cells/30 min. Decades earlier, Bierbach et al. [43] investigated the uptake and metabolism of long-chain fatty acids in isolated chicken enterocytes. Of interest was that these researchers indicated that additional glucose would depress the oxidation of fatty acids. It is established that the digestive tract has a high energy demand, but our knowledge as to which substrates supply this energy need and if this supply is subject to manipulation remains limited.

### Fat metabolism

The liver is the main site of lipid biosynthesis, or de novo lipogenesis, in broiler chickens and it is noteworthy that fasting, even for short periods of time, markedly depresses the capacity for hepatic lipogenesis [44]. Glucose is catabolized to acetyl CoA in the de novo lipogenesis pathway and acetyl CoA is then converted into fatty acids and cholesterol. Cholesterol and triacylglycerol are incorporated into very low-density lipoproteins which can enter the systemic circulation and be distributed to adipose tissues for storage [45]. Abdominal fat becomes the main fat storage site in the mature bird and this adipose tissue is largely derived from hepatic de novo lipogenesis [33]. Therefore, it is noteworthy that Goodridge and Ball [45] concluded that in pigeons as much as 96% of total body lipogenesis takes place in the liver.

### ‘Ileal brake’

In mammals, undigested nutrients in the distal small intestine, particularly fats and carbohydrates, stimulate the release of peptides which are proposed to reduce gastric emptying, increase digestibility and inhibit feed intake by enhancing satiety. This mechanism has come to be described as the ‘ileal brake’. However, the extent to which the ileal brake is operative in chickens, especially in relation to satiety, needs clarification [46]. Nevertheless, the addition of graded levels of fat to practical diets was shown to retard the transit time or the rate of passage of digesta along the gastrointestinal in laying hens by Mateos et al. [47]. The addition of 150 g/kg yellow grease to a maize-based diet delayed the initial appearance of chromic oxide in excreta by 17.6% (227 vs. 193 min) and 300 g/kg yellow grease expanded the delay to 40.4%% (271 vs. 193 min). These researchers suggested that supplemental fat may enhance the utilisation of dietary energy by slowing the rate of passage of diets, thereby generating extra-metabolic effects. Subsequently, intraluminal lipids were shown to modulate avian gastrointestinal motility in Martinez et al. [48]. The authors concluded that intraluminal infusion of lipids modulates gastrointestinal motility by decreasing the frequency of the gastric cycle, increasing duodenogastric refluxes, and elongating the migrating myoelectric complex. These actions could delay gastric emptying and increase transit time, which suggests the presence of an ‘ileal brake’ mechanism that is activated by lipids similar to that described in mammals.

### Relative abdominal fat-pad weights

The determination of relative abdominal fat-pad weights is relatively straightforward. Moreover, it is evident that abdominal fat-pad weight is a reliable indicator of total body fat content as Becker et al. [49] reported that abdominal fat constituted 20.7% and 22.8% of total fat in male and female birds, respectively. More recently, Namroud et al. [50] found that abdominal fat constituted 19.9% of total fat in 384 male birds at 28 d post-hatch. Based on the tabulated means, it may be deduced that the two parameters were highly correlated (*r* = 0.998; *P* < 0.001). Moreover, both total fat (*r* = 0.833; *P* = 0.010) and abdominal fat (*r* = 0.783; *P* = 0.021) were positively related to FCR from 10 to 28 d post-hatch across 8 dietary treatments in Namroud et al. [50]. These significant relationships only emphasise the importance of restricting the deposition of unnecessary fat to a minimum in chicken-meat production irrespective of the dietary protein regime.

Importantly, as discussed in more detail later, the anabolic properties of insulin promote glucose uptake into adipose tissue with the formation of triglycerides and hepatic lipogenesis. Hence, to some extent, insulin drives fat deposition [5].

## Proteins

Wheat typically contains more protein than maize; the mean protein concentration of 27 wheat samples was 115.5 g/kg as opposed to 80.0 g/kg in seven maize samples in one Australian survey [51]. In addition, there are differences in the amino acid profiles of the two feed grains as maize contains more leucine and alanine than wheat in absolute terms but wheat contains noticeably more glutamate as detailed in the survey. The difference in protein concentrations means that a reduced-CP, wheat-based diet will contain more non-bound amino acids (NBAA), or single, monomeric, synthetic, crystalline amino acids, than a corresponding maize-based diet. Also, the larger the dietary CP reduction, the greater NBAA inclusions will need to be to meet amino acid specifications. However, the likelihood is that the bioequivalence of non-bound and protein-bound amino acids are not identical [23]. Fundamentally, this is because NBAA have more rapid rates of intestinal uptakes than protein-bound amino acids. The digestion rate constants of non-bound *d,l-*methionine (8.49 × 10^−2^ min^−1^) and lysine HCl (8.78 × 10^−2^ min^−1^) were more rapid than the balance of protein-bound amino acids (2.35 × 10^−2^ min^−1^) by nearly a four-fold factor in broiler chickens offered standard sorghum-based diets in Liu et al. [52].

### Digestion of protein, absorption of amino acids

The digestion of proteins and absorption of amino acids in broiler chickens are relatively well identified. Protein digestion is initiated in the proventriculus where pepsin and HCl secretions split proteins into polypeptides. Several pancreatic enzymes (trypsin, chymotrypsin, carboxypeptidase, elastase) are released into the duodenum to convert polypeptides into short peptide fragments, which are, in turn, converted to di- and tri-peptides (or oligopeptides) by amino peptidase and dipeptidase in the apical membrane of enterocytes [53]. Approximately 70% to 85% of amino acids are absorbed as oligopeptides rather than as monomeric amino acids [54]. Intestinal uptakes of monomeric (neutral, basic, acidic) amino acids are conducted via an array of more than twenty Na^+^-dependent and Na^+^-independent transport systems with overlapping affinities [55]. Alternatively, intestinal uptakes of oligopeptides are conducted via the Pept-1 transporter, which has been identified in chickens [56, 57]. PepT-1 is located in the intestinal brush border membrane and is an H^+^-coupled co-transporter that requires proton binding to absorb dipeptides and tripeptides. Critically, the Pept-1 transporter functions in concert with the Na^+^/H^+^ exchanger, NHE as NHE generates the proton gradient for H^+^/peptide co-transport via PepT-1. Thus, Pept-1 is not directly Na^+^-dependent; nevertheless, NHE is reliant on the activity of the so-called ‘sodium pump’ (Na^+^/K^+^-ATPase), large numbers of which are located in the baso-lateral membrane of enterocytes. Consequently, the functions of PepT-1 and NHE are inextricably related and intestinal uptakes of oligopeptides are both rapid following digestion and energetically efficient [58–60].

Increasing quantities of NBAA are present in reduced-CP diets. Axiomatically, NBAA do not require digestion; therefore, their intestinal uptakes are more rapid than protein-bound amino acids and generate higher plasma amino acid concentrations [61]. The more rapid intestinal uptakes of NBAA are fundamental to the differences in the post-enteral bioavailability between non-bound and protein-bound amino acids, because their entries into the systemic pool lack synchronicity. The likelihood is that this dichotomy in intestinal uptake rates generates post-enteral amino acid imbalances and the adverse effects of amino acid imbalances in broiler chickens were demonstrated by Swatson et al. [62]. In this feeding study dietary amino acid balances were deliberately disturbed and, as a result, weight gain was compromised by 43.0% (29.8 vs. 17.0 g/bird/d) and feed conversion by 30.8% (2.850 vs. 2.079) from 10 to 24 d post-hatch in broilers offered 200 g/kg CP diets. Instructively, Huston and Scott [63] indicated that the adverse effects of amino acid imbalances are more readily demonstrated in birds offered low protein diets.

### Post-enteral metabolism

Protein accretion is determined by the balance between protein synthesis and protein degradation or protein turnover. The anabolic hormone, insulin, by restricting protein degradation, in particular, increases skeletal protein deposition and growth [5]. The pivotal relationship between insulin and protein turnover is discussed in more depth in Section "Insulin and reduced-CP broiler diets".

The body does not have a store for amino acids; consequently, dietary amino acids that exceed requirements for protein synthesis, are rapidly catabolised [64]. Critically, amino acid catabolism has the potential to generate elevated plasma ammonia concentrations arising from the deamination of amino acids. Schreurs et al. [65] contended that differences in intestinal uptake rates of amino acids generates post-enteral amino acid imbalances and post-enteral oxidation and deamination of surplus amino acids. The postprandial oxidative losses of egg white protein and an equivalent mix of NBAA were compared in rats using [^13^CO_2_] breath tests by Nolles et al. [66]. Postprandial oxidative losses of non-bound leucine were found to be significantly higher than protein-derived leucine. That non-bound and protein-bound amino acids are not fully bioequivalent is a real challenge because postprandial oxidation leads to elevated plasma ammonia (NH_3_) concentrations. Moreover, NH_3_ is inherently toxic, probably more so in avian species than mammals [67] and could result in ‘ammonia overload’ and compromised growth performance without adequate NH_3_ detoxification. Indeed, elevated plasma NH_3_ concentrations have been associated with compromised growth performance in broiler chickens in several studies [50, 68–70]. These outcomes are supported by the studies of Hofmann et al. [71] and Brink et al. [72].

### Ammonia detoxification

Ammonia detoxification is proficiently covered in the Stern and Mozdziak [73] review of ammonia metabolism in avian and mammalian species. Initially, NH_3_ is captured by glutamic acid in a condensation reaction which generates glutamine and is catalysed by glutamine synthetase [74]. Secondly, glutamine enters the Krebs uric acid cycle where it is converted to uric acid, which requires an input of one mole of glycine for every mole of uric acid excreted [75]. Also, NH_3_ detoxification attracts metabolic costs in terms of energy inputs. The synthesis and excretion of uric acid to void NH_3_-N in urine generates a minimal energy cost of 64.7 kJ/g N excreted as uric acid in poultry [76]. Detoxification of NH_3_ is usually adequate; however, in the event of inadequate NH_3_ detoxification the resultant NH_3_ overload compromises growth performance, which would be exacerbated by high dietary NBAA inclusions.

### Non-bound amino acid inclusions

Instructively, Baker [77] issued the caveat that there are limits to the extent that intact protein can be replaced by NBAA in terms of achieving maximal weight gain and feed efficiency. Thus, there is effectively a “ceiling” on NBAA inclusions that should not be exceeded in the formulation of reduced-CP diets, although this upper limit is probably not a constant. This topic was investigated in Macelline et al. [78] which found that protein sources manipulated growth performance in broiler chickens as defined by an equilateral-triangle response surface design. The dominant protein sources in the three apical diets were soybean meal, whey protein and NBAA. The average weight gain was 2021 g/bird (1,900 to 2,089), feed intake 2,907 g/bird (2,777 to 2,998), FCR 1.436 (1.484 to 1.401) and abdominal fat-pad weights of 10.22 g/kg (8.45 to 12.30) from 14 to 35 d post-hatch. The range of observations across the ten dietary treatments are shown in parentheses and they indicate the magnitude of the impact of different protein sources can have on growth performance. Moreover, it was deduced that the optimal NBAA inclusion rate to support broiler growth performance was 13.1 g/kg in this study. In a subsequent study, Macelline et al. [79] investigated the influence of NBAA inclusions of 12.1 and 21.1 g/kg in 210 g/kg CP diets and NBAA inclusions of 44.0 and 55.5 g/kg in 180 g/kg CP broiler on growth performance from 14 to 35 d pot-hatch. A quadratic relationship (*r* = 0.860;* P* < 0.001) between dietary NBAA inclusions and FCR was detected, which indicated that efficiency of feed conversion was compromised when NBAA exceeded 18.5 g/kg. Both these finding support the concept that there is a ceiling on NBAA inclusions, but this upper limit is not a constant.

## Starch

Starch is typically the greatest source of metabolisable energy in poultry diets and is present mainly as intact starch granules as steam-pelleting causes only limited starch gelatinisation [80]. Starch digestion in the fowl, with an emphasis on maize, was reviewed by Moran [81] and the utilisation of energy from maize starch was not considered to be limiting in poultry. Starch digestion in poultry and pigs was reviewed by Wiseman [82] with more of a focus on variations in wheat starch digestibility. This researcher suggested that starch digested at a gradual rate along the small intestine may advantage growth performance in broiler chickens and that there could be merit in separating starch into different categories including rapidly digested starch, slowly digested starch and starch that is resistant to digestion.

### Starch digestion, glucose absorption

Starch digestion and glucose absorption is quite straightforward in poultry. Starch digestion in poultry is the sole province of pancreatic α-amylase secreted into the duodenum. Apparent ileal digestibility coefficients of starch in maize-based diets ranged from 0.873 to 0.993 around a mean of 0.950 in a review of 11 studies [83]. The corresponding figures for wheat-based diets was a range from 0.790 to 0.990 across 9 studies with a mean coefficient of 0.916. In an earlier study, 38 samples of wheat grown in Australia were analysed [84]. Apparent ileal digestibility coefficients of starch ranged from 0.818 to 0.999 around a mean of 0.932. Thus, the digestibility of maize starch is generally better than wheat starch and one contributing factor could be the higher gut viscosities in birds offered wheat-based diets triggered by the presence of soluble non-starch polysaccharides in wheat [82]. It was established that the Na^+^-D-glucose cotransporter, SGLT1, is pivotal for intestinal glucose absorption in mice by Gorboulev et al. [85] and subsequently, Shibata et al. [86] reported on the development of glucose absorption from the jejunum via SGLT1 in broiler chickens. Thus, SGLT1, can be responsible for the co-absorption of both glucose and NBAA via sodium dependent pathways. This common route of absorption raises the distinct possibility of competition between starch/glucose and protein/amino acids for intestinal uptakes via co-absorption with sodium.

### Starch digestion rates

That the starch digestion rates along the small intestine of broiler chickens differ among feedstuffs was demonstrated by Weurding et al. [87]. The in vitro digestion rate of wheat starch (0.035/min) is far more rapid than either maize (0.017/min) or sorghum (0.018/min) based on determinations of 37 unprocessed samples in Giuberti et al. [88]. Also, wheat (29.5%) contained more rapidly digestible starch than maize (20.9%) or sorghum (16.2%) and wheat (65.8) had a higher glycaemic index than maize (39.5) or sorghum (15.9). In broiler chickens, Liu et al. [89] found the starch digestion rate of wheat-based diets was 34.3% (10.62 vs. 7.91 × 10^−2^ min^−1^) more rapid than maize-based diets. It was subsequently reported that wheat-based diets (0.117) supported a higher starch digestion rate constant then diets based on either maize (0.086) or sorghum (0.075) in broiler chickens [90].

Moreover, starch digestion rates have been shown to influence broiler growth performance [91–93]. Rapidly digested wheat starch was compared with slowly digested pea starch in starter, grower and finisher broiler diets which contained soybean meal as a protein source in Herwig et al. [94]. A blend of 25% slow (pea) starch and 75% rapid (wheat) starch supported the minimum FCR and improved breast-meat yield at 31 d post-hatch. Therefore, the provision of some slowly digestible starch advantaged bird performance presumably by diluting the rapid starch digestion rate of wheat; thus, the implication is that the digestion rate of wheat starch is excessively rapid. The more rapid starch digestion rate in wheat than maize is not the only factor differentiating the feed grains. However, reduced-CP diets typically contain more starch, and potentially glucose, than standard diets, which would amplify any impacts differences in starch digestion rates have on broiler growth performance.

### Glycaemic indices

Classically, starches have been classified as rapidly digestible starch (RDS), slowly digestible starch (SDS), or resistant starch (RS) in human nutrition, based on the rate and extent of their digestibility or their glycaemic index [95]. Based on in vitro data, the starch fractions are defined as follows. RDS is the amount of glucose released after 20 min, SDS is the amount of glucose released between 20 and 120 min of hydrolysis and RS is the total starch minus the amount of glucose released during the in vitro procedure. It may be anticipated that RDS would be absorbed in the jejunum, SDS mainly in the ileum and RS would be metabolised in the hindgut in broiler chickens. Glycaemic indices are not used widely in poultry nutrition; however, Liu et al. [19] did evaluate resistant maize starch in broiler chickens. It was found that feeding broilers with diets containing increasing concentrations of resistant starch impaired small intestinal development resulting in lower apparent total tract retention of nutrients and poorer growth performance and carcass traits of broiler chickens.

### Competition between glucose and amino acids for intestinal uptakes

The likelihood of competition effectively between starch and protein or glucose and amino acids for intestinal uptakes has been considered [96, 97]. Subsequently, Stevens et al. [98] discussed the advances the intestinal transport of amino acids and sugars made using membrane vesicles and Vinardell [99] reviewed the mutual inhibition in the intestinal uptakes of sugars and amino acids. More recently, Moss et al. [100] reduced dietary CP concentrations by partially replacing maize grain with maize starch. Thus, the control diet had an analysed starch concentration of 269 g/kg; whereas, the five dietary treatments had an average starch concentration of 436 g/kg. These unusually high starch concentrations were derived from both maize grain and maize starch per se. Apparent digestibility coefficients of starch and 16 amino acids were determined in four segments of the small intestine. Significant negative correlations were detected between starch and 9 amino acids in the distal jejunum, 12 amino acids in the proximal ileum and 11 amino acids in the distal ileum. Interestingly, a total of 8 amino acids (arginine, histidine, isoleucine, methionine, phenylalanine, threonine, valine, glutamate) were negatively correlated with starch in the three small intestinal segments. Moss et al. [100] suggested that these negative relationships could be attributable to competition between amino acids and glucose for co-absorption with sodium for their intestinal uptakes via their respective Na^+^-dependent transport systems.

### Starch concentrations in reduced-CP diets

Finally, the typically elevated starch concentrations in reduced-CP broiler diets, of itself, may be impeding progress [101]. Elevated starch concentrations and, in turn, high plasma glucose concentrations could be increasing hepatic de novo lipogenesis and fat deposition to the detriment of growth performance [10]. In two studies, in both wheat-based [102] and maize-based [103] broiler diets ‘capping’ or condensing dietary starch:protein ratios has shown some promise. In the maize-based study, capping starch:protein ratios in 175 g/kg CP diets significantly improved weight gain by 3.45% (2,398 vs. 2,318 g/bird) and feed conversion ratio by 3.75% (1.360 vs. 1.413) and relative abdominal fat-pad weights were numerically lighter by 10.3% (11.47 vs. 12.78 g/kg). Dietary starch:protein ratios were condensed from 2.76 to 2.35 in the 175 g/kg CP diets largely by replacing 99 g/kg soybean meal with 165 g/kg full-fat soy which allowed a reduction in maize inclusions from 613 to 500 g/kg and analysed starch concentrations from 505 to 368 g/kg.

## Insulin and reduced-CP broiler diets

Insulin plays a pivotal role in glucose homeostasis, but insulin also promotes the synthesis of protein, fat and carbohydrate; therefore, insulin may be regarded as an anabolic hormone [104]. In humans, it appears that insulin enhances protein accretion primarily by attenuating protein degradation rather than increasing protein synthesis and the effects of insulin on amino acid and protein metabolism from an historical perspective have been reviewed [105]. However, the mechanisms of insulin action and insulin resistance in mammalian species are extraordinarily complex as reflected in the comprehensive review of Petersen and Shulman [106]. The speculative perception that the feed grain basis of reduced-CP diets, specifically either maize or wheat, modifies the anabolic impact of insulin on the growth performance of broiler chickens was advanced by Selle et al. [105]. This section considers this proposition in more detail.

The importance of insulin in broiler chickens is usually overlooked, or even dismissed, because avian species are considered both hyperglycaemic and resistant to insulin [107, 108]. Birds do maintain physiologically elevated plasma glucose levels, which are 1.5 to 2 times higher than those in similarly sized mammals such as rabbits and rats. Plasma glucose concentrations averaged 233 mg/dL in male and 219 mg/dL in female broiler chickens over a 24-h cycle [109]. However, this persistent hyperglycaemia is not normally associated with any pathological outcomes in avian species [110]. Insulin resistance has been defined as an attenuated biological response to circulating insulin levels or impaired sensitivity to insulin mediated glucose disposal [111]. Shiraishi et al. [112] suggested that insulin resistance exists in the central nervous system (CNS) of broiler chicks, more so than in layer chicks, this was attributed to hyperinsulinemia down-regulating CNS insulin receptor expression.

However, it was concluded by Seki et al. [113] that boiler chickens lack the insulin-responsive glucose transporter GLUT-4 and increasing concentrations of GLUT-4 at the plasma membrane, triggered by insulin, regulates glucose transport into cells. However, Tokushima et al. [114] investigated glucose uptakes in skeletal muscles of insulin-injected chicks and found that in vivo glucose transport across the plasma membrane in most skeletal muscles tested, other than cardiac muscle, was increased by insulin administration to chicks. They concluded that despite the intrinsic lack the GLUT-4 homologous gene, the insulin-responsive glucose transporter in mammals, an insulin-responsive glucose transport mechanism is present in chickens.

It is noteworthy that the avian tissues used in the Seki et al. [113] study were taken from 28-d old Ross 308 birds. This is because there are indications that young broiler chickens are sensitive to insulin and that resistance to the hormone develops in older birds [115]. In addition, Tokushima et al. [116] characterised insulin-glucose interactions in newly hatched broiler chicks. They concluded that these birds are highly sensitivity to insulin and are under the control of specific insulin-glucose interactions. Also, these researchers found that 60 min after administration a 40 μg/kg insulin injection reduced plasma glucose concentrations by approximately 54% in day-old chicks and by 39% in 21-day-old chicks, which is consistent with the concept that broiler chickens develop insulin resistance with age.

The dismissive view of insulin in broilers may stem in part from Langslow et al. [117], where it was concluded that the regulation and role of plasma insulin concentration in chickens is very different from that in rat and man. This is at odds with Simon and Rosselin [118] who contended that insulin regulation and pancreatic β-cell function in the chicken and in mammals are qualitatively similar. Subsequently, Simon [119] expressed the opinion that it is very likely that the role of insulin in birds has been underestimated and then argued that the conservative, dismissive view of insulin in poultry is no longer tenable [120]. Subsequently, Scanes [121] indicated that insulin plays an important role in the control of carbohydrate and lipid metabolism in poultry as insulin increases the storage of energy as glycogen in liver and muscles and triglyceride in adipose tissue. Elevated circulating glucose concentrations were observed after fed chickens received insulin antisera in Simon et al. [122]. Indeed, these researchers concluded that their results strongly suggested that in fed chickens, plasma glucose is mainly, if not exclusively, controlled by plasma insulin.

Clearly marked differences in opinions exist as to the importance of insulin in broiler chickens and without further research, the role of insulin in avian species will remain a conundrum [123]. Nevertheless, in this review our prime interest is the differences between maize and wheat in the context of reduced-CP diets. Therefore, it is relevant that Maurice and Jensen [124] demonstrated that maize- and wheat-based diets diversely influenced fat metabolism in laying hens and adult Japanese quail more than 40 years ago. Maize-based diets generated numerically heavier relative abdominal fat-pad weights by 27.1% (53.0 vs. 41.7 g/kg) in laying hens than wheat-based diets. Overall, de novo lipogenesis and hepatic lipid accumulation were significantly higher in birds offered maize-based diets. The researchers concluded that the differences in hepatic lipid accumulation due to maize- versus wheat-based diets are related to changes in rates of lipogenesis. It is then relevant that Dimitriadis et al. [104] indicated that insulin increases triglyceride uptakes from the blood into adipose tissue and stimulates fatty acid and triacylglycerol synthesis, which suggests that the more pronounced responses observed by Maurice and Jensen [124] may have been driven by insulin.

Two studies are of interest in that they suggest insulin does play a physiological role in broiler chickens. Remarkably, a single 400 μg/kg administration of insulin to 1-day-old chicks significantly increased body weights by 3.54% (935 vs. 903 g/bird) at 21 d and by 3.07% (3,631 vs. 3,523 g/bird) at 50 d post-hatch and increased relative abdominal fat-pad weights by 8.70% (25 vs. 23 g/kg) in Sato et al. [125]. It is noteworthy that these researchers found that insulin enhanced cell proliferation in chicken myoblasts. In the second study, glucose and insulin plasma concentrations in chickens selected high or low body weights were compared by Sinsigalli et al. [126]. Chickens were intubated with 2.0 g/kg glucose following a 24 h fast and plasma and blood was collected at 20 min intervals up to 100 min post administration at 21, 42, 63 and 84 d post-hatch. Average plasma glucose and insulin concentrations in heavier birds exceeded their lighter counterparts by 20.7% (292 vs. 242 mg/100 mL) and a 2.5-fold factor (220 vs. 89 pg/mL), respectively, at 42 d-post hatch. However, there was a positive linear relationship (*r* = 0.935; *P* = 0.006) between plasma glucose and insulin concentrations in heavy birds, which was not apparent (*r* = 0.346; *P* = 0.501) in lightweight birds. It was concluded that excessive fat deposition in chickens developed through selection for high body weight appears to be associated with impaired glucose tolerance, mild or moderate hyperinsulinemia, hyperglucagonemia and perhaps insulin resistance.

There is a paucity of studies where plasma concentrations of glucose and insulin have been determined in applied feeding studies with broiler chickens. In Kulcsar et al. [127], starter, grower and finisher broiler diets with an average CP content of 200 g/kg and energy density of 13.0 MJ/kg were based on either maize or wheat. Blood samples were taken from birds with access to feed at 42 d post-hatch. Plasma insulin concentrations were 27.4% (12.1 vs. 9.5 mIU/L) higher in birds offered maize-based diet and maize-based diets supported fractionally higher plasma glucose concentrations by 2.63% (11.7 vs. 11.4 mmol/L) than wheat-based diets. Three butyrate treatments were applied to the control maize and wheat diets and across all eight maize-based diets supported significantly (*P* = 0.001) higher plasma insulin levels, but glucose levels were not significantly different. Subsequently Kulcsár et al. [128], in a second maize vs. wheat comparison, found that different cereal types could influence the expression of key insulin signaling proteins in broilers which they attributed to differences in the caecal production of short chain fatty acids, particularly butyrate.

Plasma concentrations of glucose and insulin were determined in broiler chickens offered either maize- or wheat-based diets with standard and reduced-CP concentrations at 7, 21 and 42 d post-hatch by Petrilla et al. [129]. Dietary CP concentrations averaged 207 or 176 g/kg across the starter, grower and finisher diets and the determined glucose and insulin plasma concentrations are shown in Table 2. The pattern of plasma glucose concentrations was similar in maize- and wheat-based diets although at 42 d post-hatch plasma glucose fell by 6.88% in maize-based diets as opposed to a fractional increase of 0.70% percent in wheat-based diets, which meant that the wheat birds had higher plasma glucose concentrations by 4.20% (14.39 vs. 13.81 mmol/L) at 42 d post-hatch. Plasma insulin concentrations were similar between the two feed grains at 21 d post-hatch. However, at 7 d post-hatch insulin concentrations increased by 5.25% in wheat-based diets but decreased by 6.39% in maize-based diets following the dietary CP reduction. In contrast, at 42 d post hatch plasma insulin concentrations increased by 16.7% in maize-based diets but decreased by 15.9% in wheat-based diets. Thus, birds offered maize-based, reduced-CP diets had 27.1% (6.081 vs. 4.783 ng/mL) higher plasma insulin concentrations than their wheat-based counterparts. Collectively, these outcomes are consistent with the proposal that young chicks are sensitive to insulin and with the suggestion that broilers offered reduced-CP, maize-based diets are more sensitive to insulin than their wheat-based counterparts. Many factors could be responsible for this difference but, while speculative, the slower digestion rate of starch in maize than wheat generating a more sustained insulin release is one candidate.

**Table 2:** Plasma concentrations of glucose (mmol/L) and insulin (ng/mL) in broiler chickens offered maize- or wheat-based diets with average CP concentrations of 207 or 176 g/kg at 7, 21 and 42 d post-hatch. Adapted from Petrilla et al. [129]

Item	Maize-based diets			Wheat-based diets		
	207 g/kg	176 g/kg	Difference	207 g/kg	176 g/kg	Difference
Glucose						
7 d	14.07	18.58	32.1%	12.64	15.95	26.2%
21 d	15.69	18.84	0.96%	13.34	13.38	0.30%
42 d	14.83	13.81	−6.88%	14.29	14.39	0.70%
Mean	14.86	16.08	8.21%	13.42	14.57	8.57%
Insulin						
7 d	4.071	3.811	−6.39%	3.926	4.132	5.25%
21 d	8.377	8.567	2.27%	9.169	9.123	−0.50%
42 d	5.209	6.081	16.7%	5.688	4.783	−15.91%
Mean	8.886	6.153	4.54%	6.261	6.013	−3.96%

**Fig. 3:**
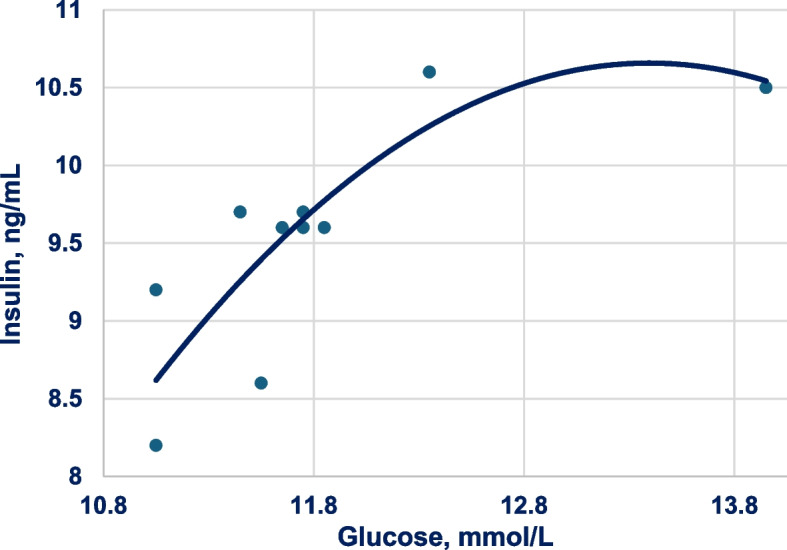
Quadratic relationship (*r* = 0.832; *P* = 0.016) between plasma concentrations of glucose and insulin in broiler chickens offered standard diets containing 150 g/kg starch derived from either cassava or maize at 0, 30, 60, 120 and 150 min post-prandial, where *y* = 8.181 × glucose − 0.306 × glucose^2^ – 45.761. Adapted from Luo et al. [130]

Support for this argument is provided by Luo et al. [130] who compared starch from maize or cassava where starch from both sources were incorporated into standard broiler diets at 150 g/kg. Importantly, cassava is a more rapidly digestible starch than maize starch [87]. At 21 d post-hatch birds were fasted for 4 h and then fed for 30 min and blood samples were at 30, 60, 90 and 120 min post-prandially to determine glucose and insulin plasma concentrations. Cassava starch generated significantly higher plasma glucose concentrations than maize starch at 30 and 60 min post-prandium. Insulin plasma concentrations peaked at 30 min with cassava starch, whereas insulin levels from diets containing maize starch increased steadily overtime and finally remained at a stable level after 60 min post-prandium. Moreover, there was a quadratic relationship (*r* = 0.852; *P* = 0.011) between plasma concentrations of glucose and insulin in broiler chickens in the Luo et al. [130] study as shown in Fig. 3.

Interestingly, against a control diet, graded levels (0, 66.7, 133.3, 200 g/kg) of resistant maize starch were included in standard, maize-soy starter and grower diets at the expense of maize starch in Liu et al. [131]. This study was discussed briefly in the previous section. The increase from 0 to 200 g/kg resistant starch linearly increased plasma glucose concentrations by 24.0% (11.46 vs. 9.24 mmol/L; *P* < 0.001) at 21 d post hatch and by 17.3% (10.79 vs. 9.20 mmol/L; *P* < 0.001) at 42 d post hatch. Similarly, the increase in resistant starch linearly increased plasma insulin concentrations by 60.7% (11.31 vs. 7.04 μIU/mL; *P* = 0.006) at 21 d post hatch and by 14.4% (12.74 vs. 11.14 μIU/mL; *P* = 0.191) at 42 d post hatch. Moreover, overall plasma glucose and insulin concentrations were linearly related (*r* = 0.957; *P* = 0.011) at 42 d post hatch.

### Dietary CP reductions – elevated plasma NH_3_ concentrations

The likelihood is that reduced-CP broiler diets can trigger elevated plasma NH_3_ concentrations or ‘ammonia overload’ from the deamination of surplus amino acids. However, elevated plasma NH_3_ concentrations can influence the metabolic fates of glucose and insulin [132].

Uric acid concentrations in excreta from the Chrystal et al. [8] study, proportions of uric acid-N to total N in excreta in birds offered the 165 g/kg CP, maize-based diet were significantly lower by 10.6 percentage units (27.4% vs. 38.0% *P* = 0.00057) than their wheat-based counterparts from retrospective determinations reported by Selle et al. [133]. The likelihood is that the underperforming birds offered the 165 g/kg CP, wheat-based diets had elevated plasma NH_3_ concentrations or ‘ammonia overload’. It is then relevant that proportions of uric acid-N to total excreta N were linearly related to weight gain (*r* = −0.587; *P* = 0.010), feed intake (*r* = −0.526; *P* = 0.025), and FCR (*r* = 0.635; *P* = 0.005) such that they were associated with significantly depressed growth performance.

It is noteworthy that Clifford et al. [134] indicated that ammonia intoxication caused derangements of intermediary carbohydrate metabolism and subsequently Visek [132] reviewed the effects of NH_3_ with an emphasis on glucose metabolism and insulin action. There are indications in mammalian species that elevated NH_3_/NH_4_^+^ plasma levels may depresses pancreatic insulin secretions and may increase insulin resistance. For example, Feldman and Lebovitz [135] found that NH_4_Cl inhibited insulin secretion stimulated by glucose in pancreatic tissue taken from golden hamsters. The impacts of NH_3_ on glucose and insulin in rats were investigated by Schlienger et al. [136] and Schlienger and Imler [137]. In the first paper, the researchers concluded that the glucose intolerance induced by ammonium acetate infusions may be due to direct effect of NH_4_^+^ on the pancreas. In the second paper, their opinion was that the abnormalities in glucose tolerance noted with elevated plasma NH_3_ levels could be partially attributed to increased insulin resistance. The metabolic and functional effects of NH_4_^+^ in rat pancreatic islets were investigated by Sener et al. [138]. They reported NH_4_^+^ caused a dose related inhibition of insulin release induced by 16.7 mmol/L glucose in isolated pancreatic islet cells, which was both rapid and reversible. Sufficient ammonium salts were infused into dogs to provoke NH_3_ toxicity in Strombeck et al. [139]. This induced hyperglycemia, hyperglucagonemia and hyperinsulinemia in intact dogs plus decreases in plasma concentrations of glutamine, alanine, threonine, glycine, lysine, valine, proline, serine, arginine, leucine, isoleucine, and methionine. Ammonium acetate was included in diets for rats at 30 g/kg, which were fed ad libitum for 7 d in Leontowicz et al. [140]. The dietary addition of ammonium acetate depressed plasma insulin concentrations by 16.5% (45.4 vs. 54.4 mU/L). It was reported by Bessman et al. [141] that NH_3_ inhibited insulin stimulation of the Krebs cycle. Insulin failed to cause any stimulation of the incorporation of succinate carbons into hepatocyte protein in the presence of NH_4_Cl. The researchers concluded that NH_3_ diminution of the Krebs cycle may affect basal mitochondrial function and responsivity to insulin and may have serious anabolic implications.

### Dietary CP reductions – metabolic acidosis

The relevance of this section is that low-grade metabolic acidosis has been described as a driver of insulin resistance in humans [142]. The principle that metabolic acidosis interferes with glucose homeostasis and insulin functionality in humans is supported in several papers and the effects of systemic and local acidosis on insulin resistance and signaling were reviewed in [143]. These researchers suggested that a strong relationship exists between acidosis and insulin metabolism or insulin receptor signaling. Metabolic acid loads have been associated with impaired glucose homeostasis and insulin resistance because metabolic acidosis interferes with intracellular insulin signaling pathways [144]. Finally, Hayata et al. [145] contended that low extracellular pH is involved in the pathogenesis of insulin resistance in skeletal muscle cells.

The impact of dietary CP reductions on the acid‐base balance in weaner pigs was investigated by Lin et al. [146]. A moderate dietary CP reduction from 185 to 165 g/kg reduced pH (7.04 vs. 7.42;* P* = 0.015) and significantly decreased HCO_3_^–^ (28.44 vs. 31.29 mmHg), pCO_2_ (37.04 vs. 39.44 mmHg), and the base excess (BE: 6.70 vs. 8.13 mmol/L) in arterial blood, which are all signs indicative of metabolic acidosis. The moderate 20 g/kg dietary CP reduction was achieved by increasing maize (596 to 656 g/kg), decreasing soybean meal (220 to 150 g/kg) and NBAA inclusions were increased from 4.24 to 12.86 g/kg. However, the dietary electrolyte balance (DEB) of soybean meal (478.6 mEq/kg) comfortably exceeds that of maize (71.5 mEq/kg) as reported by Sumathi et al. [147]. Thus, without appropriate adjustments, dietary CP reductions will cause DEB to decline and Waldroup [148] suggested that low DEB may be contributing to the poor performance of birds offered reduced-CP diets. Moreover, it is relevant that low DEB diets are associated with more acidic pH and metabolic acidosis and high DEB have the reciprocal effect [149]. The catabolism of amino acids in broilers offered reduced-CP diets, particularly methionine, cysteine and cationic amino acids, has an acidifying impact as they generate endogenous acid (H^+^) production [150]. Catabolism of one mole of methionine generates two moles of acid as per the following equation from the Patience [151] review:

$$\mathrm{2methionine}+15{\mathrm{O}}_{2}\to \mathrm{urea}+9\mathrm{C{O}}_{2}+7{\mathrm{H}}_{2}\mathrm{O}+4{\mathrm{H}}^{+}+\mathrm{S{{O}}_{4}}^{2-}$$

Increased lysine HCl inclusions have an acidifying effect as one gram of lysine·HCl increases dietary acid levels by 7 mEq/kg [151]. Interestingly, lysine·HCl has been used to treat metabolic alkalosis in human patients [152]. In the Lin et al. [146] study, dietary inclusions of lysine HCl were increased from 3.0 to 7.0 g/kg and *d,l*-methionine from 1.0 to 2.0 g/kg with the CP reduction as part of the overall increase in NBAA from 4.24 to 12.86 g/kg which, coupled with the partial substitution of soybean meal with maize, would have contributed to a lower DEB and the observed signs of metabolic acidosis.

Given the association between low DEB and metabolic acidosis it is noteworthy that Chrystal et al. [153] compared 156 g/kg CP, maize-based diets, with DEB of either 120 or 230 mEq/kg. The lower 120 mEq/kg DEB significantly depressed apparent jejunal digestibilities of six amino acids (arginine, isoleucine, leucine, methionine, valine, proline) by an average of 3.40% (0.766 vs. 0.793), but growth performance was not impacted. Given that Johnson and Karunajeewa [154] recommended a DEB between 250 and 300 mEq/kg in standard starter (220 g/kg CP) and finisher (185 g/kg CP) broiler diets, the DEB values in the Chrystal et al. [153] comparison may have been too conservative.

Metabolic acidosis accelerates protein degradation rates by stimulating the ubiquitin–proteasome pathway in rats [155], which would have a negative impact on protein turnover. Moreover, amino acid metabolism and acid–base homeostasis are closely intertwined in animal nutrition [151]. Therefore, the compromised amino acid digestibilities in Chrystal et al. [153] are of interest. Also, NH_3_ metabolism plays a critical role in acid–base homeostasis [156]. In the event of metabolic acidosis, glutamine is extracted and catabolised in the kidneys for the generation of NH_4_^+^ ions to facilitate the excretion of HCO_3_^−^ ions and acids in the urine. The renal extraction of glutamine is balanced by glutamine release from muscle and liver coupled with less glutamine catabolism in the gut mucosa. Thus, glutamine is pivotal to acid–base homeostasis as metabolic acidosis is compensated by a net release of HCO_3_^−^ arising from the renal metabolism of glutamine [157]. Tissue glutamine concentrations have been shown to correlate with net protein turnover and there is evidence that glutamine may both stimulate protein synthesis and inhibit protein degradation [158]. However, any positive impacts of glutamine may be indirect because Han et al. [159] concluded that glutamate converted from glutamine is an essential mediator that enhances calcium signaling in the glutamine-amplifying effect on insulin secretion. It was indicated by Wu and Thompson [160] that glutamine had an overall anabolic effect in isolated avian skeletal myocytes; however, again this may reflect the positive effect of glutamine on insulin secretion.

The effects of high NBAA inclusions in diets for broiler chickens on metabolic acidosis were investigated in Ibrahim et al. [161]. In this study, a soy protein isolate was either partially or totally replaced with a blend of non-bound or free amino acids. The dietary switch from protein-bound to NBAA did significantly reduce blood pH, HCO_3_^−^ and BE, but on short-term basis. Interestingly, it had been shown earlier that the kidney in poultry can adapt to chronic metabolic acidosis [162]. However, it was found in Ibrahim et al. [163] that inclusions of glutamine and asparagine permitted higher NBAA concentrations in reduced-CP broiler diets before growth performance or nitrogen accretion was compromised. This was attributed to glutamine and asparagine compensating for challenges to acid–base homeostasis via a compensation of metabolic acidosis.

## Summary

**Fig. 4:**
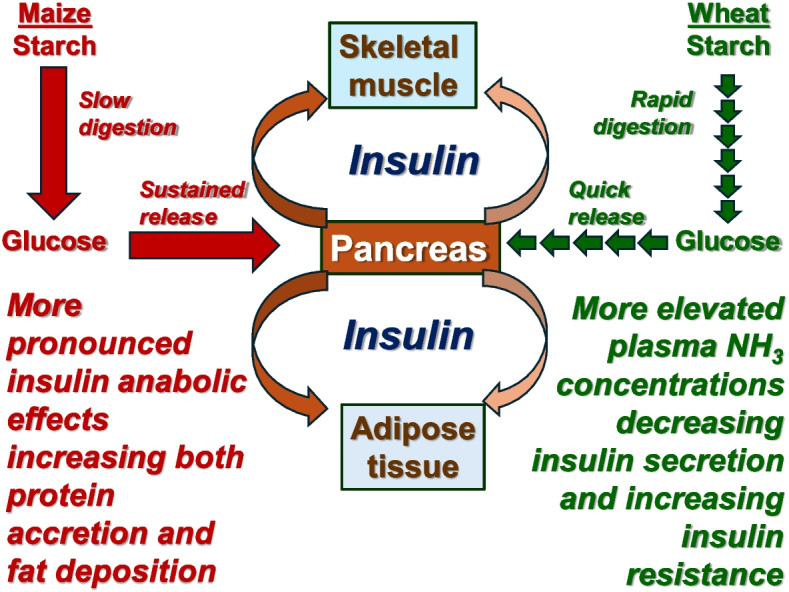
Conceptual diagram of the different impacts of starch digestion rate from maize vs. wheat on glucose absorption and pancreatic insulin secretion and, in turn, protein accretion and fat deposition in the context of reduced-CP diets

From the quest to develop reduced-CP diets, it has become increasingly evident that sources and concentrations of starch and protein/amino acids influence both fat deposition and broiler growth performance. A conceptual diagram of the different impacts of maize vs. wheat starch digestion rates on glucose absorption, pancreatic insulin secretion, protein accretion and fat deposition in the context of reduced-CP diets is included as Fig. 4. The superiority of maize over wheat in the context of reduced-CP diets where maize supports enhanced weight gains and more efficient feed conversion ratios despite higher levels of fat deposition. Typically, the formulation of reduced-CP broiler diets axiomatically increases feed grain inclusions and starch concentrations, which means that the more rapid rates of starch digestion and glucose absorption from wheat than maize becomes increasingly important. While the importance of insulin in avian species is debatable, it remains plausible that insulin is involved in this dichotomy between wheat and maize in the context of reduced CP diets. This is because of the possibility that slower rates of starch digestion and glucose offered maize-based diets may trigger a more sustained insulin release. Moreover, the likelihood is that dietary CP reductions have the potential to elevate circulating NH_3_ concentrations and promote metabolic acidosis and, importantly, both elevated plasma NH_3_ concentrations and metabolic acidosis can negatively influence insulin secretion and insulin sensitivity. Thus, the anabolic properties of insulin would be more likely to promote fat deposition and protein accretion and in birds offered maize-based, reduced-CP diets than their wheat-based counterparts, which would account for the ‘intriguing paradox’ of outcomes observed in practice. There is the real possibility that the anabolic impacts of insulin influence the performance of boiler chickens offered reduced-CP diets. Judicious, sequential determinations of plasma concentrations of glucose, insulin, glucagon, NH_3_, free amino acids and lipid parameters and their relationships to growth performance and relative fat-pad weights should be included in future studies to define the importance of insulin in the development of reduced-CP diets, as suggested in Selle et al. [5]. And, as discussed in Macelline et al. [123], 3-methylhistidine could be used as a biomarker for protein degradation, which, in turn, may be indicative of the anabolic activity of insulin because insulin enhances protein turnover by supressing protein degradation.

## Data Availability

Any additional data will be made available by the Corresponding Author on request.

## Abbreviations

AME: Apparent metabolizable energy
BCAA: Branched-chain amino acids
CP: Crude protein
FCR: Feed conversion ratio
N: Nitrogen
NBAA: Non-bound amino acids
NH_3_: Ammonia
